# Nosemosis in Russian *Apis mellifera* L. Populations: Distribution and Association with Hybridization

**DOI:** 10.3390/insects16060641

**Published:** 2025-06-18

**Authors:** Milyausha Kaskinova, Luisa Gaifullina, Gleb Zaitsev, Alexandr Davydychev, Elena Saltykova

**Affiliations:** 1 Institute of Biochemistry and Genetics, Ufa Federal Research Center, Russian Academy of Sciences, Ufa 450054, Russia; kasmil08g@gmail.com (M.K.); lurim260578@gmail.com (L.G.); esaltykova1960@gmail.com (E.S.); 2 Laboratory of Forestry, Ufa Institute of Biology, Ufa Federal Research Center, Russian Academy of Sciences, Ufa 450054, Russia; shur25@yandex.ru

**Keywords:** *Apis mellifera* L., *Nosema ceranae*, *Nosema apis*, nosemosis, hybridization, *tRNAleu-COII*, SSR loci

## Abstract

One of the common causes of mass death in bee colonies is the infectious disease nosemosis, which is caused by two types of microsporidia, *Nosema apis* and *Nosema ceranae*. Of the many factors contributing to the spread of nosemosis, in this paper we consider the hybridization of subspecies of *Apis mellifera* L. In most of Russia, the native subspecies is the dark forest bee *Apis mellifera mellifera*, which is representative of the evolutionary lineage M. The export of bee packages and queens from the southern regions of Russia and other countries has led to the fragmentation of the range of these subspecies. First, we determined the maternal and paternal ancestry of 349 honey bee colonies across 12 beekeeping regions of Russia using the mitochondrial *tRNAleu-COII* locus and nine nuclear SSR markers (*Ap243*, *4a110*, *A024*, *A008*, *A43*, *A113*, *A088*, *Ap049*, and *A028*). Among them, 140 colonies belonged to subspecies *A. m. mellifera*, 58 colonies were of hybrid origin, and 151 colonies belonged to evolutionary lineage C. Then, using microscopy and PCR analysis, we performed diagnostics of nosemosis in the studied colonies: *N. apis* was detected in 87 colonies, *N. ceranae* in 102 colonies, and coinfection was observed in 36 colonies. The results of our study indicate that the main reservoir of *Nosema* microsporidia was bees of evolutionary lineage C.

## Simple Summary

*Nosema* is one of the causes of mass death in bee colonies. The wide spread of microsporidia of the genus *Nosema* is facilitated by many factors. In this study, we considered the influence of factors such as the export of bee colonies and subsequent hybridization with native subspecies of honey bees on the spread of nosemosis. First, using genetic methods, we established the origins of bee colonies from the 12 regions of Russia. Then, using microscopy and PCR analysis, we performed diagnostics of nosemosis. Our results show that the main reservoirs of *Nosema* microsporidia in Russian *Apis mellifera* populations are introduced bees of evolutionary lineage C (i.e., subspecies *A. m. carnica* and *A. m. ligustica*).

## 1. Introduction

The honey bee *Apis mellifera* L. is a versatile and regulated pollinator of flowering plants, contributing to the conservation of biodiversity in natural ecosystems and increasing the productivity of agroecosystems [1]. The high economic importance of honey bees as pollinators is complemented by various bee products used in the food industry and medicine. The honey bee is distinguished by significant geographic and subspecies diversity, with distinctive features formed by adaptations to environmental conditions. Today, about 30 subspecies of *A. mellifera* L. are known, which have been grouped into several evolutionary lineages based on morphometric and genetic data [2,3,4]. In Russia, beekeepers keep several subspecies of honey bee: *A. m. mellifera*, *A. m. caucasica*, *A. m. carnica*, and *A. m. ligustica*. Of these, the first two are native [2,5]. *A. m. mellifera* belongs to the evolutionary lineage M [2]. *A. m. caucasica* belongs to the evolutionary lineage O based on morphometric and whole-genome data [2,4], but shares common *tRNAleu-COII* haplotypes with subspecies from evolutionary lineage C [6,7]. These subspecies are not the only subspecies whose evolutionary positions have not yet been accurately established [8]. *Apis m. carnica* and *A. m. ligustica* are the most common representatives of the lineage C [4,6].

In Russia, most bee colonies (94%) are kept in private apiaries [9], and wild colonies of *A. m. mellifera* live mainly in the Burzyansky district of Bashkortostan [10]. The mass import of bee colonies from the southern regions of Russia (where the subspecies *A. m. caucasica* is native) and neighboring countries has significantly reduced the habitat of *A. m. mellifera* and led to uncontrolled hybridization [11]. Previously, *A. m. mellifera’s* range extended from the Pyrenees to the Ural Mountains [2], but now this subspecies is represented by separate populations that have survived in some European countries [12,13,14] and Russia [7,11]. Hybridization is dangerous not only because of the loss of local gene pools but also because of the spread of bee diseases [15,16].

One of the common causes of mass death in bee colonies is the infectious disease nosemosis, which is caused by *Nosema apis* and *Nosema ceranae* [17,18]. Recent investigation showed its controversial taxonomic position, but a formal redefinition by Tokarev et al. [19] was not considered valid by other researchers [20]. Therefore, in our study we used the conventional name of the genus; that is, *Nosema*. *Nosema apis* was originally restricted to Europe and North America, and *N. ceranae*, a parasite of the Chinese wax bee *A. cerana*, was restricted to Southeast Asia. However, several studies have shown that *N. ceranae* had expanded its host range [21,22]. Currently, *N. ceranae* is considered to be infective to members of the stingless bees (*Meliponini*), wasps (*Vespidae*), and some species of bumblebees and bees of the genus *Apis* [23,24,25]. In apiaries in many countries, *N. ceranae* is becoming the dominant microsporidia species [26,27,28,29,30,31,32,33,34].

The detection of *N. ceranae* in preserved *A. mellifera* specimens from the 1990s indicated that the species had been circulating among European bees for a considerable time before it was first discovered [35]. Rangel et al. (2016) [28] showed that *N. ceranae* infestation levels increased 7-fold between 1991 and 2013 in a wild Africanized honey bee population. The high frequency of infection and wide distribution of microsporidia are facilitated by: (1) high resistance of spores in the environment; (2) untimely diagnosis of the disease due to the asymptomatic development of *N. ceranae* infection at the initial stages and the absence of spores in the intestine at the intracellular stage of pathogen development; (3) trophallaxis and grooming; (4) polyandry and the possibility of sexual transmission of infection; (5) joint use of habitats of honey bees, contaminated food resources, and beekeeping equipment; (6) trading of bee colonies and bee products; and (7) the spread of *N. ceranae* among various Hymenoptera species, which may act as reservoir hosts for *N. ceranae* [18,29,36,37,38,39].

In this study, we examined whether the export of bee packages and queens (and subsequent hybridization with local bees) affects the spread of nosemosis. As mentioned above, the native subspecies in the northern regions of the country is the dark forest bee *A. m. mellifera*, whereas in the south of Russia, there is a habitat of the gray mountain Caucasian bee *A. m. caucasica* [40]. Due to hybridization, the ranges of these two native subspecies have become extremely fragmented, as most beekeepers have switched to keeping *A. m. carnica* and interbreed hybrids, because they are more accessible. We assume that the uncontrolled import of packages and queens is one of the main factors in the spread of bee diseases, in particular *N. ceranae*, in Russian populations of *A. mellifera* L. To our knowledge, no previous study has focused on identifying a link between the origin of bee colonies and the prevalence of nosemosis. In our study, we formed three samples of bees depending on their genetic origin and assessed them for the carriage of the microsporidia *N. apis* and *N. ceranae*.

## 2. Materials and Methods

### 2.1. Sampling

Bees were sampled in the Altai Territory (N = 8), Belgorod Region (N = 29), Krasnodar Territory (N = 29), Leningrad Region (N = 10), Novgorod Region (N = 17), Orenburg Region (N = 5), Republic of Adygeya (N = 26), Ryazan Region (N = 10), Samara Region (N = 3), Sverdlovsk Region (N = 12), Ulyanovsk Region (N = 1), and the Republic of Bashkortostan (N = 199). A total of 349 colonies sampled from 2022 to 2024 from 12 regions of Russia were analyzed (Supplementary Table S1).

### 2.2. Determining the Origin of Bee Colonies

The subspecies were determined using PCR analysis of mtDNA (intergenic region *tRNAleu-COII*) and SSR loci (*Ap243*, *4a110*, *A024*, *A008*, *A43*, *A113*, *A088*, *Ap049*, and *A028*) [41,42]. Total DNA was isolated from the thorax muscles using a DNA-EXTRAN-2 kit (Syntol, Moscow, Russia). The quality and quantity of total DNA were analyzed using a NanoDrop 1000 spectrophotometer (Thermo, Waltham, MA, USA).

PCR analysis of the *tRNAleu-COII* locus was performed using primers E2 5’-GGCAGAATAAGTGACATTG-3’ and H2 5’-CAATATCATTGATGAACC-3’ [43]. The primer sequences for the microsatellite loci are presented in Table S2.

The PCR mixture in a final volume of 20 μL included: 15 μL sterile ddH2O, 2 μL of 10 × PCR buffer, 0.4 μL dNTP, 0.6 μL each primer (10 pmol/μL), 0.3 μL Taq DNA polymerase (Syntol, Moscow, Russia), and 2 μL DNA template. PCR conditions for the *tRNAleu-COII* locus: initial denaturation at 94 °C for 5 min, followed by 30 cycles of denaturation at 94 °C for 30 s, annealing at 50 °C for 30 s, and elongation at 72 °C for 1 min with a final elongation at 72 °C for 10 min. The PCR conditions for microsatellite loci were similar, with the only difference being the annealing temperature of 57 °C. PCR products were examined on 8% polyacrylamide gels (PAAG) stained with ethidium bromide. The gels were visualized in a Gel Doc™ XR+ photosystem (BioRad, Hercules, CA, USA).

Data obtained from microsatellite loci were used to determine the genetic structure of the samples using Structure 2.3.4. *Apis m. mellifera* samples from the Burzyansky District of the Republic of Bashkortostan (N = 123), and Perm Territory (N = 136) were used as a reference group for the evolutionary lineage M. The samples from the Republic of Adygeya (N = 91), Krasnodar Territory (N = 120), and Uzbekistan (N = 70) were used as a reference group for evolutionary lineage C. We analyzed each studied sample from Section 2.1 separately—that is, we included five reference samples and one tested sample in the input file for the Structure 2.3.4. There were 12 program runs in total, corresponding to the number of studied regions. The number of expected clusters, K, for a given reference sample was set from 1 to 5 (by the number of reference samples). The optimal K value was calculated using Structure Selector software [44,45]. The analysis was performed using the admixture model with information about the geographic localization of samples (LocPrior) and with Burnin Period and MCMC equal to 50,000 and 100,000 replicates, respectively. The analysis results were processed in CLUMPP 1.1.2 using the FullSearch algorithm.

### 2.3. Microscopy and PCR Diagnostics of Nosemosis

Many methods have been developed for studying microsporidia, which are summarized in this work [46]. In our study, we did not count the number of spores; we only established the fact of the presence or absence of the disease. Therefore, the standard protocol was modified slightly to suit the conditions of our laboratory.

Microscopy and PCR analysis were used to diagnose nosemosis. Bee samples were stored in 96% ethanol at −30 °C. The contents of the midgut of 30 bees were extracted with tweezers and homogenized with a pestle in 3 mL of distilled water (100 μL of ddH2O per bee gut). The resulting homogenate was transferred to an Eppendorf tube and stored in a freezer at −30 °C until subsequent microscopy and DNA extraction.

**Figure 1:**
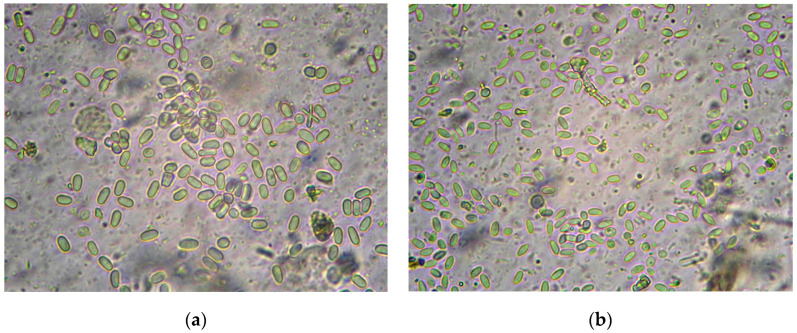
*Nosema* spp. spores from the *Apis mellifera* intestinal homogenate: (**a**) *N. apis*; (**b**) *N. ceranae*, (×400).

For microscopy, 10 µL of homogenate was used. Figure 1 shows spores of *N. apis* and *N. ceranae* (Levenhuk MED Series 4GL-M microscope, Tampa, FL, USA, magnification 400×). In photos of the same scale, differences in the size of the spores and their shape are noticeable.

**Figure 2:**
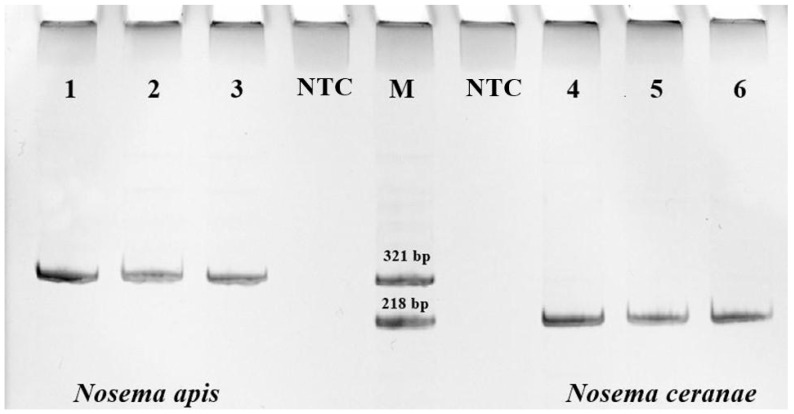
PCR analysis of DNA from the midgut of honey bees using primers specific for *Nosema apis* (321 bp) and *Nosema ceranae* (218 bp), where 1–6 are studied samples, NTC is “no template control”, and M denotes markers.

For DNA extraction, 300 μL of the homogenate was centrifuged for 2 min at 13,000 rpm. The supernatant was removed, and the sediment was used for further DNA extraction using a DNA-extran-2 kit (Syntol, Moscow, Russia). To purify DNA from pigments and other impurities, the solution was additionally centrifuged in spin columns (2 min, 13,000 rpm). The quality and quantity of total DNA were analyzed using an Implen NANOPHOTOMETER N60 spectrophotometer (Munich, Germany). For PCR analysis, primers specific for *N. apis* (product size 321 bp) and *N. ceranae* (218 bp) were used [47]. To visualize the amplification products, electrophoresis in 8% polyacrylamide gel (PAGE) was used (Figure 2), followed by detection in the Gel Doc™ XR+ photosystem (BioRad, Hercules, CA, USA). The association between Nosema prevalence and bee origin was assessed using the Pearson χ^2^ coefficient in RStudio Version 1.4.1717.

## 3. Results

### 3.1. Genetic Structure of Apis mellifera Populations

In the amplification of the *tRNAleu-COII* locus, PCR products of approximately 600, 800, and 1000 bp in size were obtained, corresponding to variants Q, PQQ, and PQQQ. Allelic variants P(Q)_n_ are markers of the origin of bees from the M evolutionary lineage, allelic variant Q—from the evolutionary lineages C and O on the maternal line. Analysis of the *tRNAleu-COII* locus revealed that 198 of the 349 colonies originated from the M lineage. Of these, 140 colonies had a proportion of the M gene pool at the nuclear DNA level greater than or equal to 88%. We accepted these colonies as “pure” *A. m. mellifera* (group “m”), whereas the remaining 58 colonies were classified as hybrids (group “h”). 151 colonies belonged to the C lineage on the maternal line (group “c”). We analyzed colonies from the C lineage using only the *tRNAleu-COII* mtDNA locus because our goal was to identify hybrid colonies from the M lineage.

**Figure 3:**
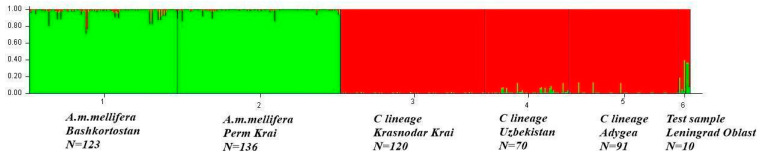
Genetic structure of the reference (No 1–5) and test (No 6) samples at K = 2.

Below is a graph showing the genetic structure of the reference sample. Each vertical line represents a separate sample, and the color indicates the probability that the individual belongs to a particular cluster. The optimal value of K was K = 2 (Delta K = 2035.33820, Figure S1). One cluster was formed by samples of *A. m. mellifera* from the Republic of Bashkortostan (Burzyan population) and Perm Krai (Perm population). The second cluster included two samples from the southern regions of Russia (Krasnodar Territory and Adygeya) and a sample from Uzbekistan. Each studied sample (sample 6 in Figure 3 shows the sample from Leningrad Region as a test sample) was analyzed separately with the reference samples (Table S1, Figure S1). Among the studied samples, colonies with a gene pool share of M over 88% were identified in the Altai Territory (4 of 8 colonies), Belgorod Region (6 of 29 colonies), Sverdlovsk Region (7 of 12 colonies), and the Republic of Bashkortostan (123 of 199 colonies). The structures of the studied samples for K = 3, 4, and 5 are shown in Figure S1.

### 3.2. Evaluation of the Relationship Between the Prevalence of Nosemosis and the Origin of Bees

To avoid the formation of a dependent sample, we used samples that were initially selected for monitoring subspecies affiliation and did not have obvious signs of disease (or we had no information about the state of the apiaries).

We used two methods for diagnosing nosemosis: microscopy and PCR analysis. PCR analysis was used to detect nosemosis in apiaries in 11 of the 12 surveyed regions. *N. ceranae* was detected in 102 colonies, and *N. apis* in 87 colonies using PCR analysis. Spores were detected in 98 samples (28% of the total number of colonies) using microscopy, whereas nosemosis was diagnosed in 153 colonies (43.8% of the total number of colonies) using PCR analysis. Therefore, in 55 colonies, nosemosis was in an active or covert phase when the spore level was below the threshold of microscopic detection. This result confirmed the importance of PCR diagnostics of nosemosis.

To assess the relationship between the prevalence of nosemosis and genetic origin of honey bees, three groups of bees were formed by origin (Table 1): 151 colonies from evolutionary lineage C (designated as “c”, allelic variant Q), 140 colonies of *A. m. mellifera* (“m”, share of gene pool M ≥ 0.88 and allelic variant P(Q)_n_), and 58 colonies of hybrid origin (“h”, share of gene pool M < 0.88 and allelic variant P(Q)_n_). The column headed Nosemosis shows the number of colonies with *Nosema*, regardless of the species.

Of the 12 regions, nosemosis was not detected in the Altai Territory (Table 2). In the Republic of Bashkortostan, 34.7% of the studied colonies were affected by nosemosis (Table S3). The territory of the Burzyansky District is part of a specially protected territories where bee packages are prohibited. However, out of 33 selected colonies, five originated from evolutionary lineage C, and in one of them, both *N. apis* and *N. ceranae* were found.

The Pearson test was performed for three variants of bee groups by origin (Table 3). There were no significant differences in the prevalence of *N. ceranae* and *N. apis* in colonies of different origins. A possible reason for this is that nosemosis has long been established in the apiaries. However, a statistically significant relationship was found between the number of colonies affected by nosemosis and the origin of the bees. Therefore, we assume that hybrid colonies and colonies from evolutionary lineage C have a higher nosemosis load. A relationship was also found between the prevalence of *N. ceranae* in group c when comparing groups m and c without considering hybrid colonies (*p*-value = 0.05644, Table S4).

## 4. Discussion

In this study, we assessed in which of the evolutionary lineages nosemosis was most widespread and thus tried to establish the main source of *Nosema* sp. distribution. *Nosema apis* and *N. ceranae* are widespread in various regions of Russia. They were found in apiaries in Western and Eastern Siberia, in areas with a moderate and sharply continental climate [17,48,49,50,51]. We assumed that the import of bee packages and queens was one of the factors in the spread of bee diseases; in particular, *N. ceranae* in Russian populations of *A. m. mellifera*. We analyzed bee populations of different subspecies for the presence of *Nosema* spores and DNA. As a result, we found that nosemosis predominated in colonies from the evolutionary lineage C (*p*-value = 0.004633). Therefore, it can be assumed that imported colonies from the lineage C are the source of the spread of nosemosis in the population of the dark forest bee in Russia. In Russia, the most commonly introduced representatives of the evolutionary lineage C are *A. m. carnica* and *A. m. ligustica*.

The problem of preserving *A. mellifera* subspecies from hybridization has prompted scientists from different countries to search for reliable subspecies identification methods. Our laboratory has tested the method developed by Garnery et al. [43]. This method is based on the analysis of polymorphisms in the *tRNAleu-COII* intergenic locus of mitochondrial DNA and allows determination of the origin of bees along the maternal line. This simple, reliable, and inexpensive method has been proven by researchers worldwide [52,53,54,55]. However, using this method, it is impossible to differentiate *A. m. caucasica* from the C lineage subspecies and assess the influence of drone background. These issues can be addressed using microsatellite [12,42,56] and whole-genome [4,57] data. To determine the level of hybridization of colonies, we used microsatellite markers. From the microsatellite loci described by Solignac et al. [42], we selected the loci that showed the greatest differentiating ability for the samples of *A. m. mellifera*, *A. m. caucasica*, and *A. m. carnica* collected in their natural habitats in Russia [11,58]. A set of nine microsatellite loci allowed us to differentiate the subspecies *A. m. mellifera* from *A. m. caucasica* and *A. m. carnica*, but not the last two subspecies from each other. Using this method, it was found that dark forest bee populations mainly survived in the Volga Federal District [11,59]. Among the European subspecies, the dark forest bee has suffered the most from hybridization [52,60,61]. Hybridization is dangerous not only because of the loss of local gene pools but also because of the spread of bee diseases [15,16].

Several articles analyzed the role of *N. ceranae* in the mortality of honeybees and bumblebees [27,62,63,64,65]. Despite numerous studies demonstrating the negative impact of *Nosema* on the health and viability of honey bees, as well as a detailed description of the pathogenesis of the infection, the issue of the death of bee colonies due to nosemosis remains controversial. Thus, a multivariate statistical analysis of data obtained over 15 years showed that the main cause of winter colony losses in Germany was *V. destructor*, whereas *N. ceranae* infection was statistically significantly correlated with colony losses, but with no or low biological relevance [66]. A mathematical model of the relationship between nosemosis and forager losses demonstrated that *N. ceranae* causes the death of bee colonies only along with another negative factor [67]. Using RT-qPCR analysis, *N. ceranae* was detected in 96% of bees from healthy apiaries in southwestern Germany [68]. However, there are frequent reports of bee colony deaths caused by *N. ceranae* infection [64,69,70]. An acute form of nosemosis with short-term deaths of entire apiaries was observed in Kazakhstan in 2012 and 2015 [71]. A recent laboratory experiment in caged bees demonstrated a threefold increase in the mortality of insects infected with *N. ceranae* [18]. Every year, our research group receives increasing numbers of complaints from beekeepers about the death of bees, and as we have found out, most bees die from *Nosema ceranae* (unpublished data).

To combat nosemosis, timely and accurate diagnostics are necessary. In our study, we found nosemosis occurred in 16% of the colonies at the covert stage of development; i.e., microsporidia spores were not detected. Traver and Fell (2011) [31] reported that 51.1% of colonies with no spores detected by microscopy were affected by nosemosis when analyzed by PCR. Problems with diagnosing nosemosis and differentiating *Nosema* species using conventional light microscopy methods are associated with minor morphological differences between *N. apis* and *N. ceranae* spores; with the peculiarities of the *Nosema* sp. life cycle, including intracellular development at the initial stage of infection in the absence of mature spores in the intestinal lumen; as well as with the asymptomatic development of *N. ceranae* infection at the initial stages. Standard polymerase chain reaction or real-time PCR can overcome these difficulties [22,47,72].

An important aspect of preserving local subspecies is the search for surviving populations and their further protection. In Russia, the only protected population is the Burzyan population of the dark forest bee. However, we see that hybridization processes are still occurring in this population. This is facilitated by migratory beekeeping (usually these are *A. m. carnica* colonies, since they are more accessible), lack of availability in the market for packages and queens of *A. m. mellifera*, and catching swarms of unknown origin, among others. High demand for bee colonies and bee products stimulates uncontrolled import of bees of unknown subspecies and undiagnosed diseases into the territory of the Republic of Bashkortostan. This situation threatens the preservation of the subspecies *A. m. mellifera*, and the biodiversity of the natural ecosystems of the region as a whole.

## 5. Conclusions

Uncontrolled crossing of honey bee subspecies as a result of import of bee colonies or queens from other regions and countries leads to a decrease in stability and productivity, as well as the loss of the gene pool of native honey bee populations. However, the main danger lies in the spread of bee diseases [73,74,75]. The results of our research confirmed that the main reservoirs of nosemosis infection are bees of evolutionary lineage C. The detection of microsporidia in protected populations of dark forest bees demonstrates the out-of-control spread of *N. ceranae*. We hope that dissemination of information about nosemosis and the importance of preserving the native subspecies of *Apis mellifera* L. among beekeepers will help stop this process.

## Figures and Tables

**Table 1:** Nosemosis in the studied groups of honey bees.

Number of Colonies						
Group	Number of Colonies	*N. apis*	*N. ceranae*	Nosemosis	Coinfection	Microscopy
c	151	43	52	81	14	49
h	58	14	17	23	8	16
m	140	30	33	49	14	33
Total	349	87	102	153	36	98

**Table 2:** Genetic origin and prevalence of nosemosis in the regions of Russia.

Region	M mtDNA	C mtDNA	M ≥ 0.88 nDNA	*N. apis*	*N. ceranae*	Coinfection
Altai Territory (N = 8)	8	0	4	0	0	0
Belgorod Region (N = 29)	6	23	6	7	14	2
Krasnodar Territory (N = 29)	0	29	0	11	9	4
Leningrad Region (N = 10)	10	0	0	1	3	0
Novgorod Region (N = 17)	0	17	0	3	9	2
Orenburg Region (N = 5)	0	5	0	3	1	0
Republic of Adygeya (N = 26)	0	26	0	19	7	6
Ryazan Region (N = 10)	0	10	0	1	3	0
Samara Region (N = 3)	0	3	0	0	2	0
Sverdlovsk Region (N = 12)	10	2	7	1	3	0
Ulyanovsk Region (N = 1)	0	1	0	1	1	1
Republic of Bashkortostan (N = 199)	164	35	123	40	50	21
Summary (N = 349)	198	151	140	87	102	36

**Table 3:** Pearson’s chi-squared test in honey bee groups by origin.

Group	*N. apis* (%)	*N. ceranae* (%)	Nosemosis (%)
c = 151	43 (28%)	52 (34%)	81 (54%)
h = 58	14 (24%)	17 (29%)	23 (40%)
m = 140	30 (21%)	33 (24%)	49 (35%)
Pearson’s chi-squared test	X-squared = 1.9517, df = 2, *p*-value = 0.3769	X-squared = 4.1467, df = 2, *p*-value = 0.1258	X-squared = 10.749, df = 2, *p*-value = 0.004633

## Data Availability

The original contributions presented in the study are included in the article/Supplementary Material. Further inquiries can be directed to the corresponding author.

## Supplementary Materials

The following supporting information can be downloaded at https://www.mdpi.com/article/10.3390/insects16060641/s1. Table S1: Origin of bee colonies and prevalence of nosemosis in the regions of Russia (N = 349 colonies). Full version; Table S2: Primer sequences for SSR loci; Table S3: Origin of bee colonies and prevalence of nosemosis in the Republic of Bashkortostan; Table S4: Pearson’s chi-squared test in honey bee groups by origin; Figure S1: Delta K for a different number of subpopulations (on the left) and genetic structure of studied populations for K = 3–5 (on the right).
