# Supplementary material for: Nosemosis in Russian Apis mellifera L. Populations: Distribution and Association with Hybridization
Source: Insects. 2025 Jun 18;16(6):641. doi: 10.3390/insects16060641 (PMC12193922; doi:10.3390/insects16060641)
Supplement: Supplementary file 1 [file insects-16-00641-s001.zip › Suppl. Figure S1_edited.pdf]

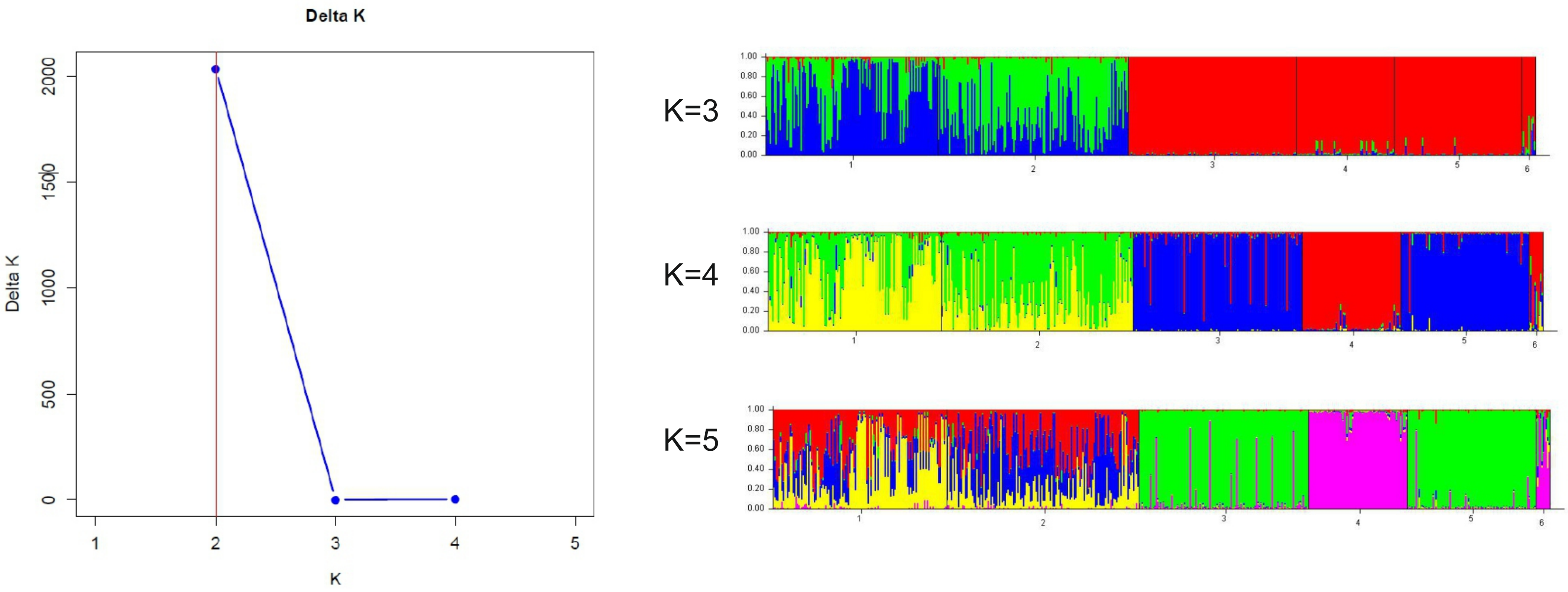

Figure S1 Delta K for a different number of subpopulations (on the left) and genetic structure of studied populations for K=3-5 (on the right).

Decoding of sample numbers in the plots on the right:
1 - reference sample from the Burzyansky District of the Republic of Bashkortostan (123 colonies)
2 - reference sample from the Perm Territory (136 colonies)
3 - reference sample from the Krasnodar Territory (120 colonies)
4 - reference sample from the Uzbekistan (70 colonies)
5 - reference sample from the Republic of Adygeya (91colonies)
6 - tested sample from Leningrad Oblast (10 colonies)
